# Supplementary material for: Comprehensive Comparative Analysis and Development of Molecular Markers for Dianthus Species Based on Complete Chloroplast Genome Sequences
Source: Int J Mol Sci. 2022 Oct 19;23(20):12567. doi: 10.3390/ijms232012567 (PMC9604191; doi:10.3390/ijms232012567)
Supplement: Supplementary file 1 [file ijms-23-12567-s001.zip › Supplementary figures S1-S4.pdf]

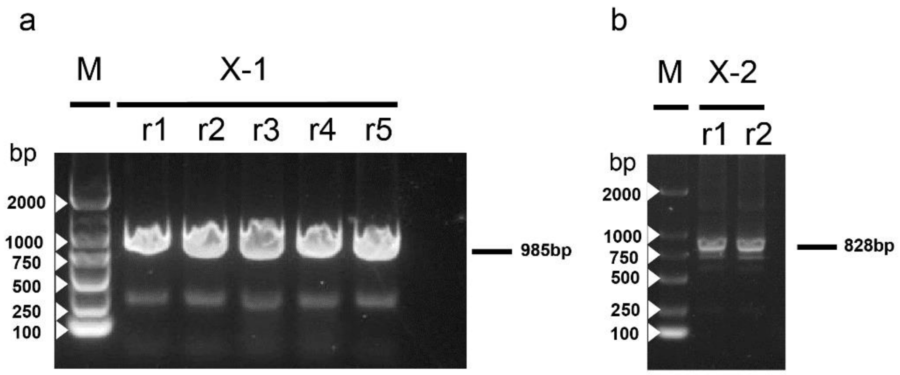

Figure S1. The gel electrophoresis results of the amplification of gaps in Dch 'X' sample using designed primers. (a) The result of X-1. r1-r5 were five repetitions. (b) The result of X-2. r1-r2 were two repetitions. Lane M was the marker of DL2000.

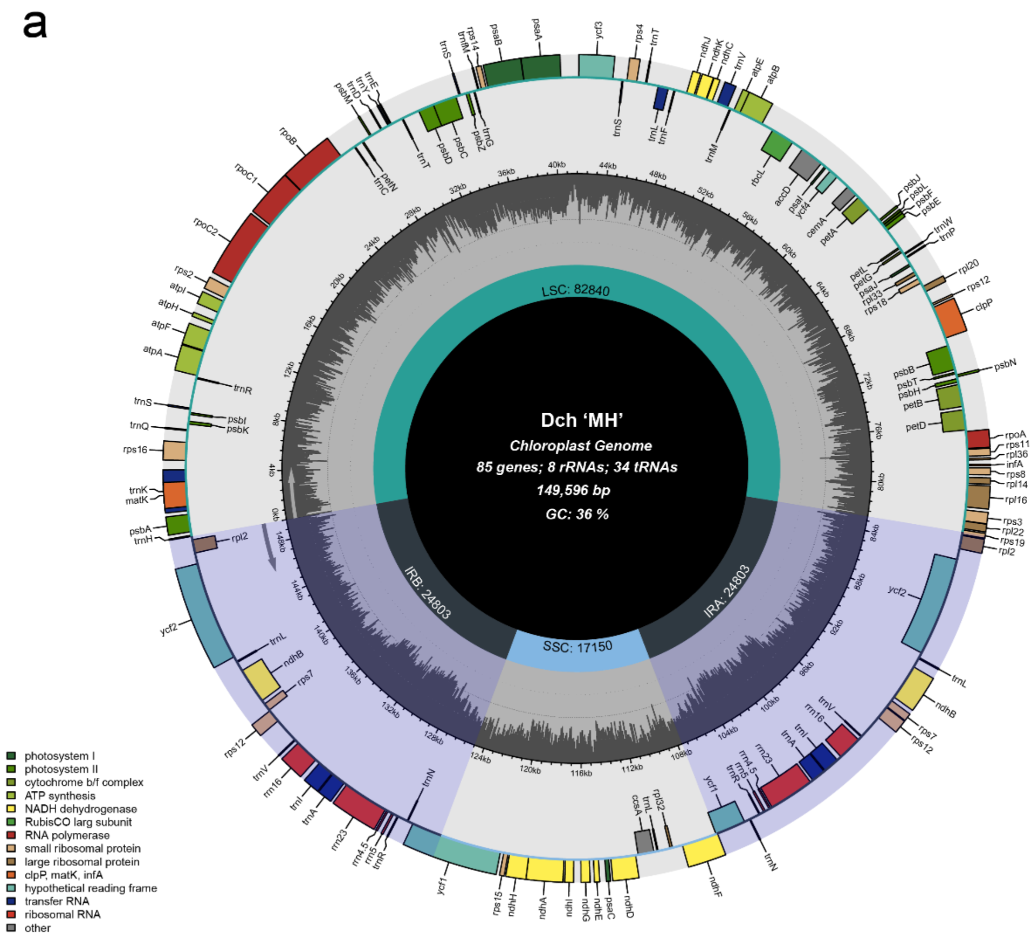

b

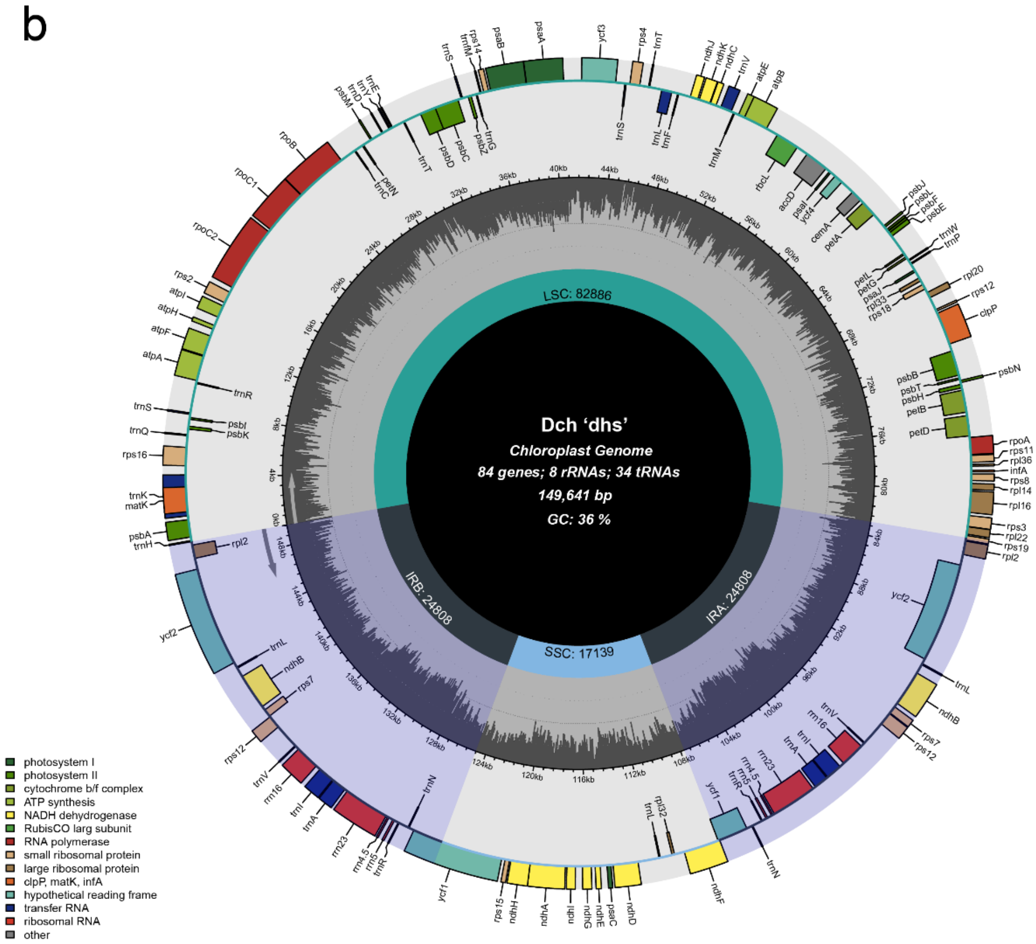

Dch ‘dhs’
Chloroplast Genome
84 genes; 8 rRNAs; 34 tRNAs
149,641 bp
GC: 36 %
LSC: 82886
IRB: 24808
IRA: 24808
SSC: 17139
photosystem I
photosystem II
cytochrome b/f complex
ATP synthesis
NADH dehydrogenase
RubisCO larg subunit
RNA polymerase
small ribosomal protein
large ribosomal protein
clpP, matK, infA
hypothetical reading frame
transfer RNA
ribosomal RNA
other

c

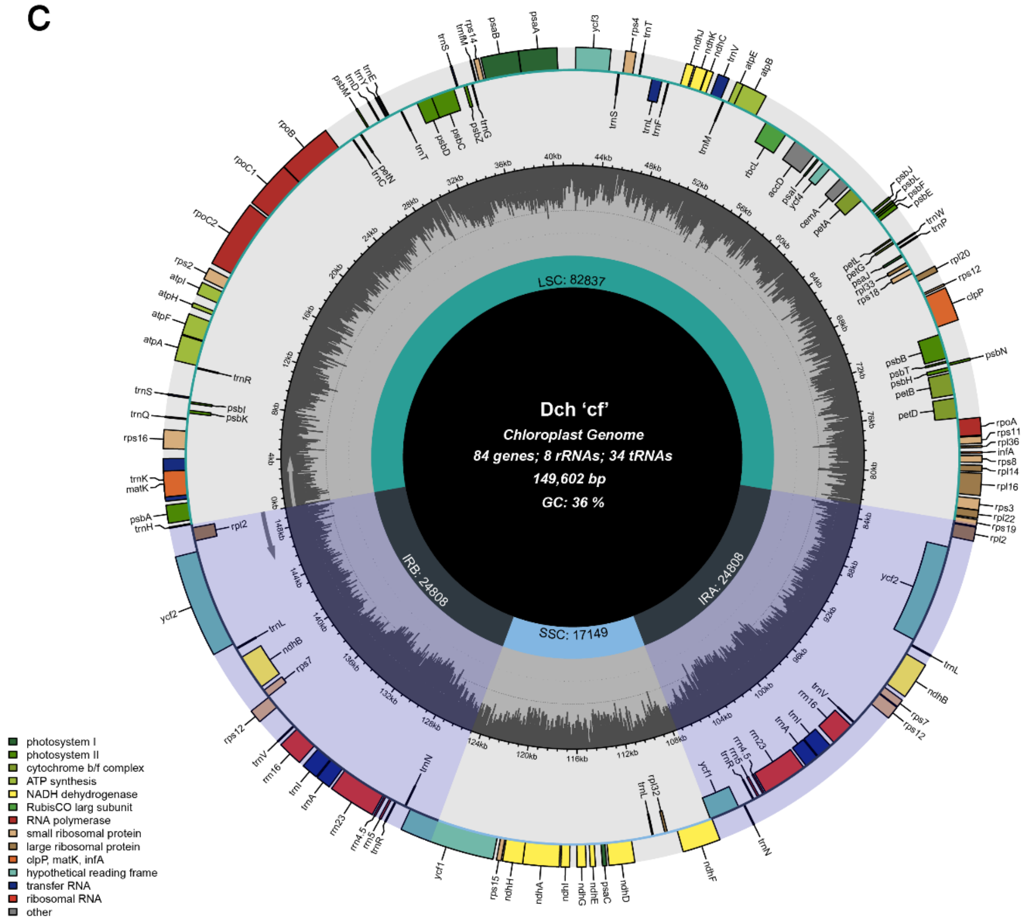

Dch ‘cf’
Chloroplast Genome
84 genes; 8 rRNAs; 34 tRNAs
149,602 bp
GC: 36 %
LSC: 82837
IRB: 24808
IRA: 24808
SSC: 17149
photosystem I
photosystem II
cytochrome b/f complex
ATP synthesis
NADH dehydrogenase
RubisCO larg subunit
RNA polymerase
small ribosomal protein
large ribosomal protein
clpP, matK, infA
hypothetical reading frame
transfer RNA
ribosomal RNA
other

d

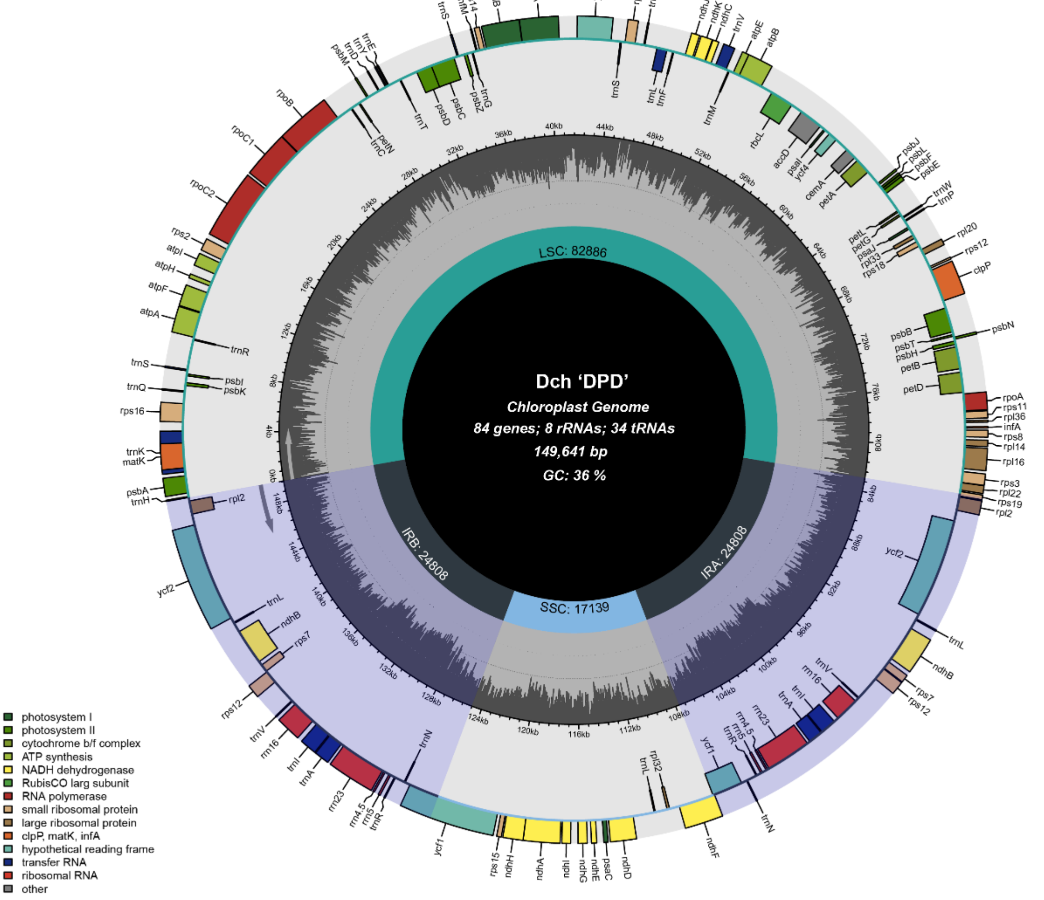
Dch 'DPD'
Chloroplast Genome
84 genes; 8 rRNAs; 34 tRNAs
149,641 bp
GC: 36 %
LSC: 82886
IRA: 24808
SSC: 17139
IRB: 24808
photosystem I
photosystem II
cytochrome b/f complex
ATP synthesis
NADH dehydrogenase
RubisCO larg subunit
RNA polymerase
small ribosomal protein
large ribosomal protein
clpP, matK, infA
hypothetical reading frame
transfer RNA
ribosomal RNA
other

e

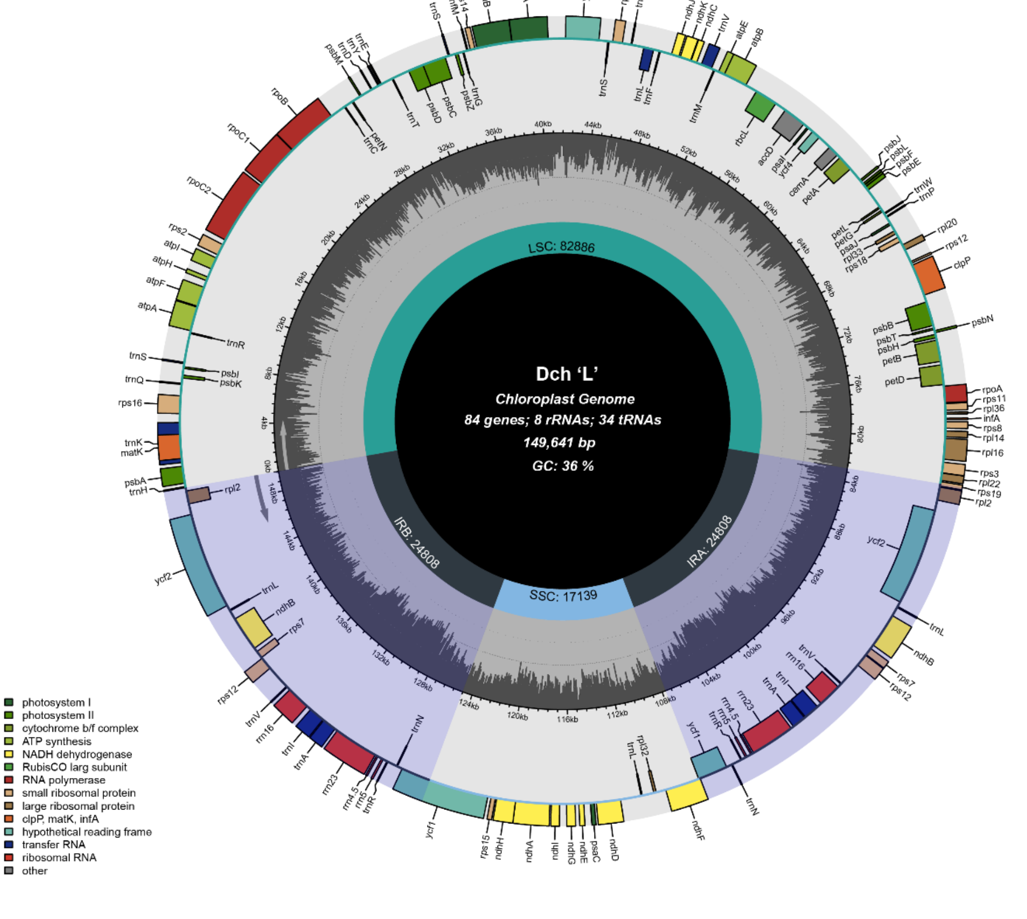
Dch 'L'
Chloroplast Genome
84 genes; 8 rRNAs; 34 tRNAs
149,641 bp
GC: 36 %
LSC: 82886
IRA: 24808
SSC: 17139
IRB: 24808
photosystem I
photosystem II
cytochrome b/f complex
ATP synthesis
NADH dehydrogenase
RubisCO larg subunit
RNA polymerase
small ribosomal protein
large ribosomal protein
clpP, matK, infA
hypothetical reading frame
transfer RNA
ribosomal RNA
other

f

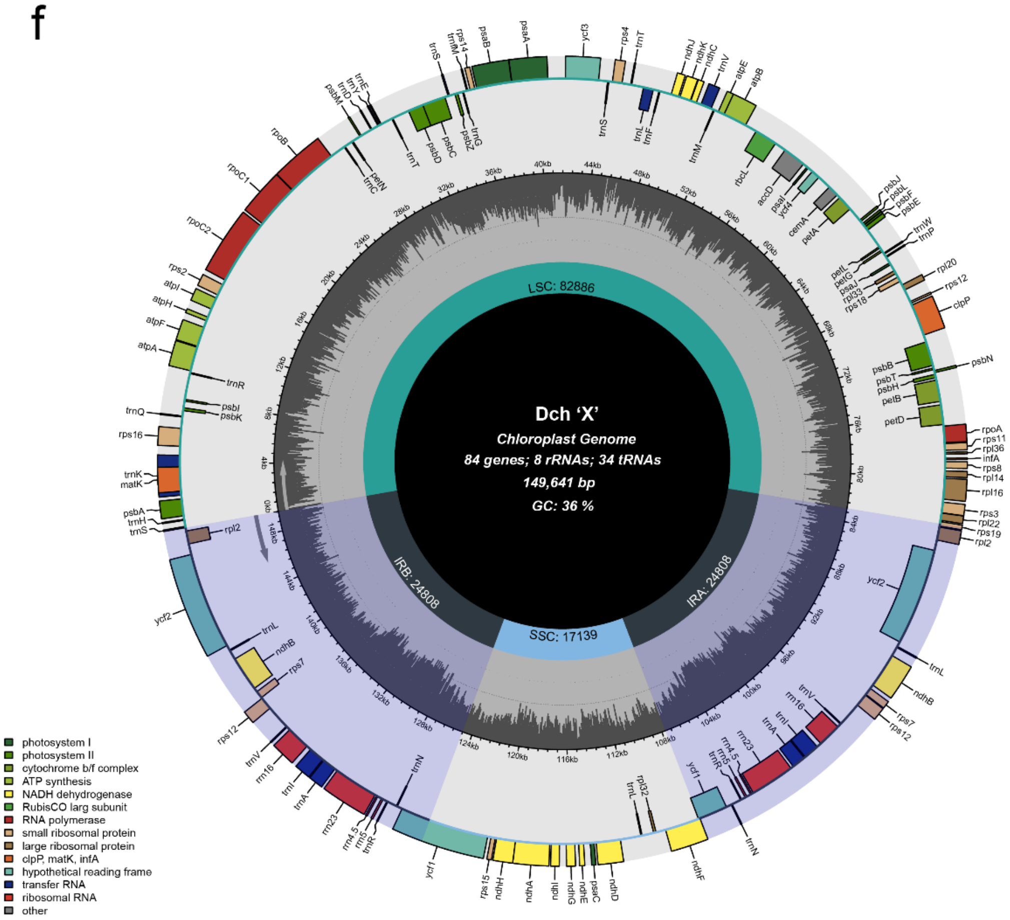
Dch 'X'
Chloroplast Genome
84 genes; 8 rRNAs; 34 tRNAs
149,641 bp
GC: 36 %
LSC: 82886
IRB: 24808
IRA: 24808
SSC: 17139
photosystem I
photosystem II
cytochrome b/f complex
ATP synthesis
NADH dehydrogenase
RubisCO larg subunit
RNA polymerase
small ribosomal protein
large ribosomal protein
clpP, matK, infA
hypothetical reading frame
transfer RNA
ribosomal RNA
other

g

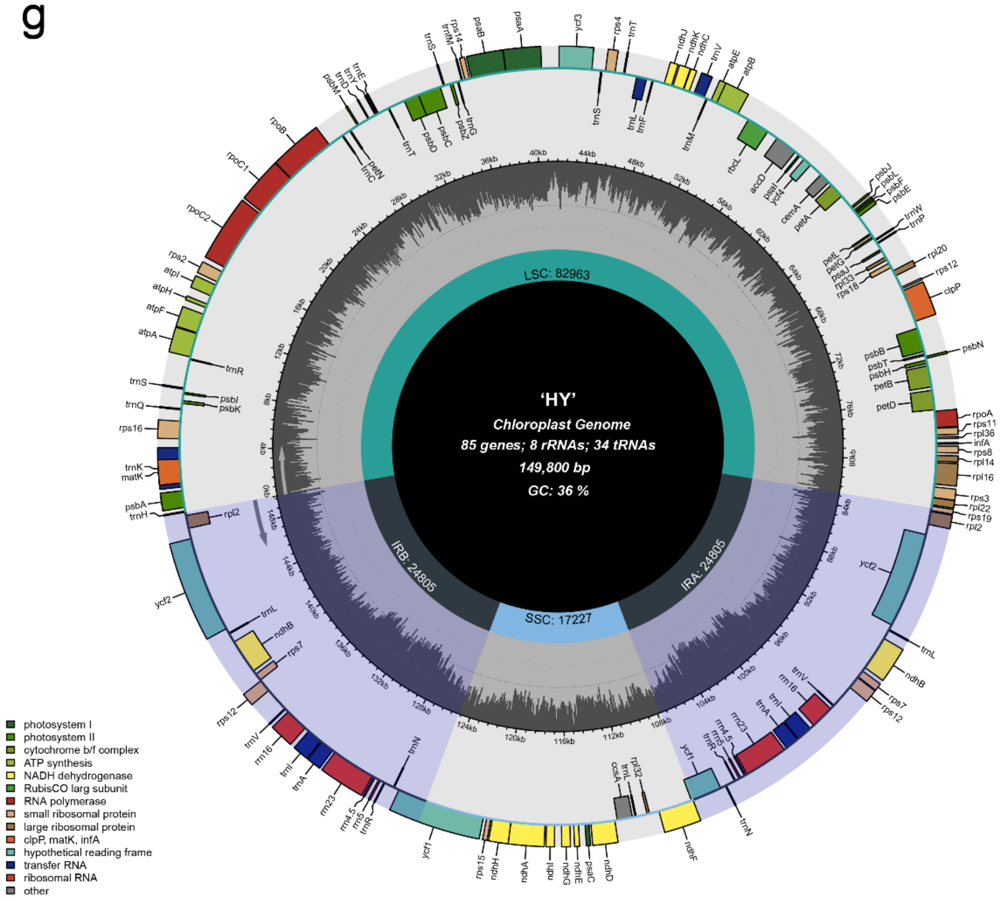
'HY'
Chloroplast Genome
85 genes; 8 rRNAs; 34 tRNAs
149,800 bp
GC: 36 %
LSC: 82963
IRB: 24805
IRA: 24805
SSC: 17227
photosystem I
photosystem II
cytochrome b/f complex
ATP synthesis
NADH dehydrogenase
RubisCO larg subunit
RNA polymerase
small ribosomal protein
large ribosomal protein
clpP, matK, infA
hypothetical reading frame
transfer RNA
ribosomal RNA
other

h

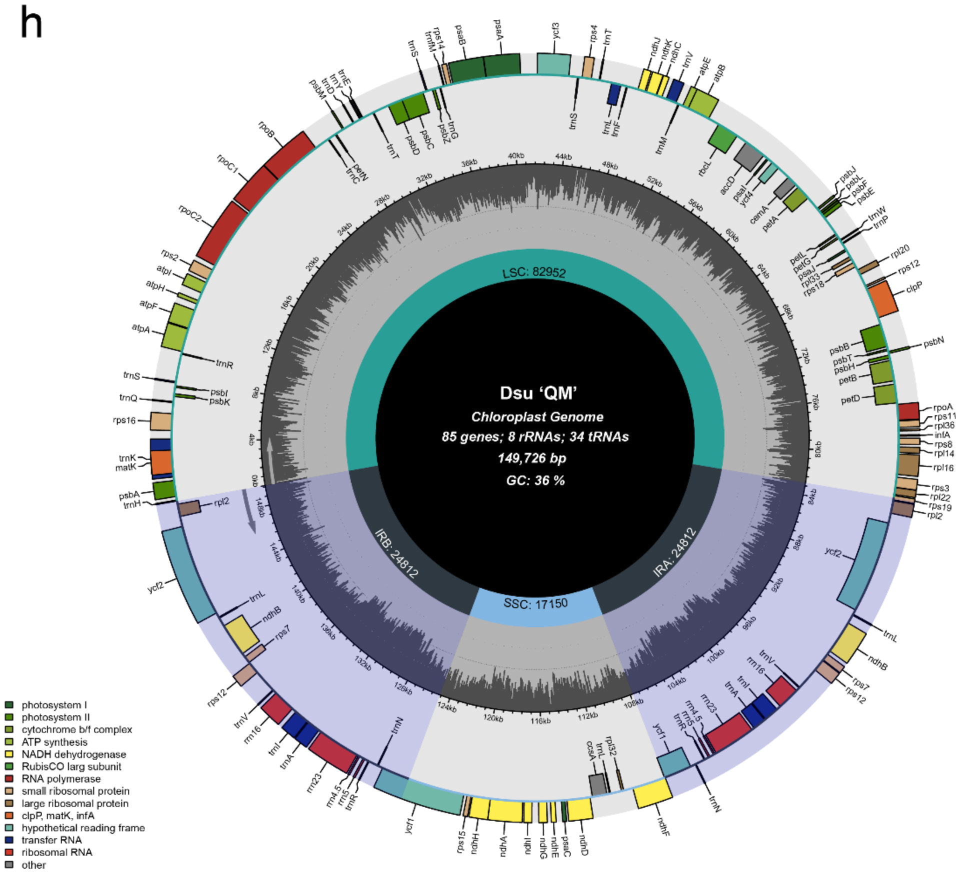
LSC: 82952
Dsu ‘QM’
Chloroplast Genome
85 genes; 8 rRNAs; 34 tRNAs
149,726 bp
GC: 36 %
IRB: 24812
IRA: 24812
SSC: 17150
photosystem I
photosystem II
cytochrome b/f complex
ATP synthesis
NADH dehydrogenase
RubisCO larg subunit
RNA polymerase
small ribosomal protein
large ribosomal protein
clpP, matK, infA
hypothetical reading frame
transfer RNA
ribosomal RNA
other

i

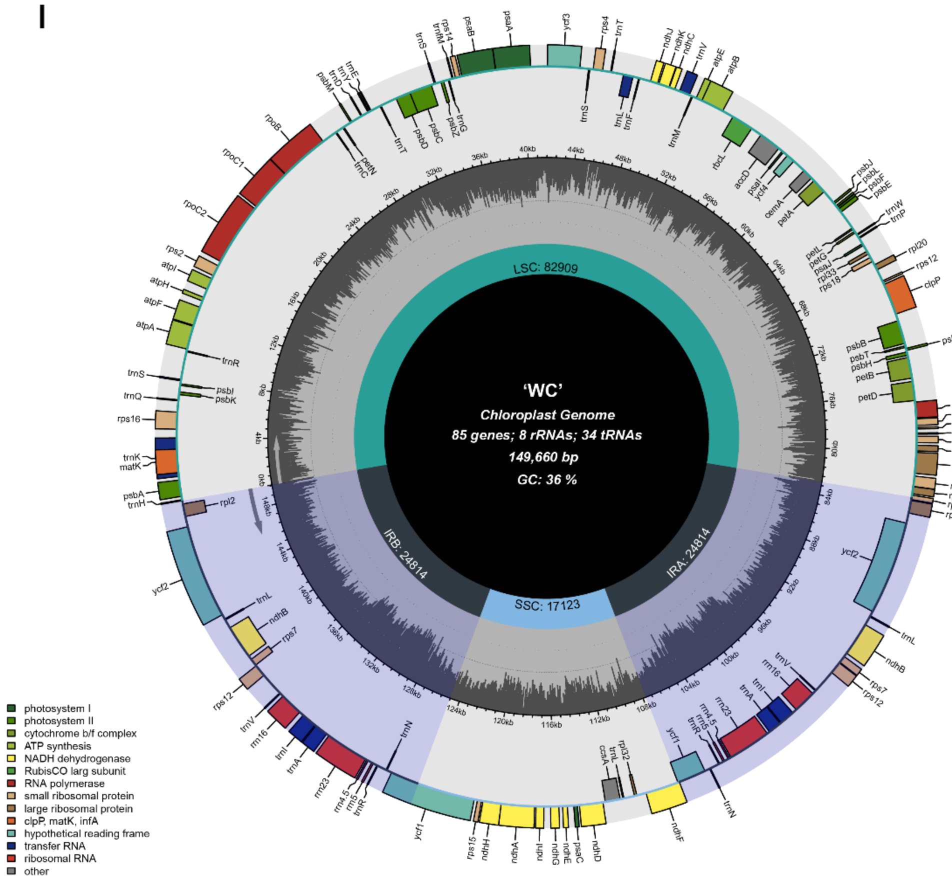
LSC: 82909
‘WC’
Chloroplast Genome
85 genes; 8 rRNAs; 34 tRNAs
149,660 bp
GC: 36 %
IRB: 24814
IRA: 24814
SSC: 17123
photosystem I
photosystem II
cytochrome b/f complex
ATP synthesis
NADH dehydrogenase
RubisCO larg subunit
RNA polymerase
small ribosomal protein
large ribosomal protein
clpP, matK, infA
hypothetical reading frame
transfer RNA
ribosomal RNA
other

j

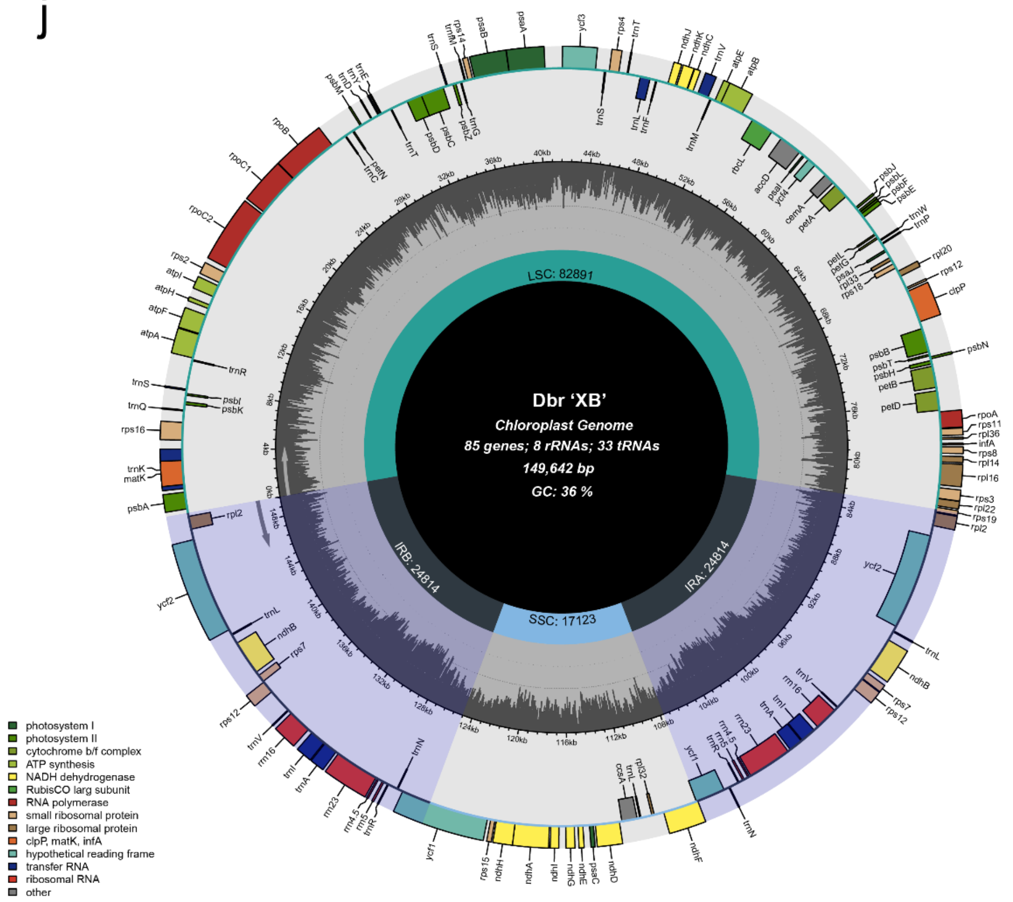
Dbr 'XB'
Chloroplast Genome
85 genes; 8 rRNAs; 33 tRNAs
149,642 bp
GC: 36 %
LSC: 82891
IRA: 24814
SSC: 17123
IRB: 24814
photosystem I
photosystem II
cytochrome b/f complex
ATP synthesis
NADH dehydrogenase
RubisCO larg subunit
RNA polymerase
small ribosomal protein
large ribosomal protein
clpP, matK, infA
hypothetical reading frame
transfer RNA
ribosomal RNA
other

k

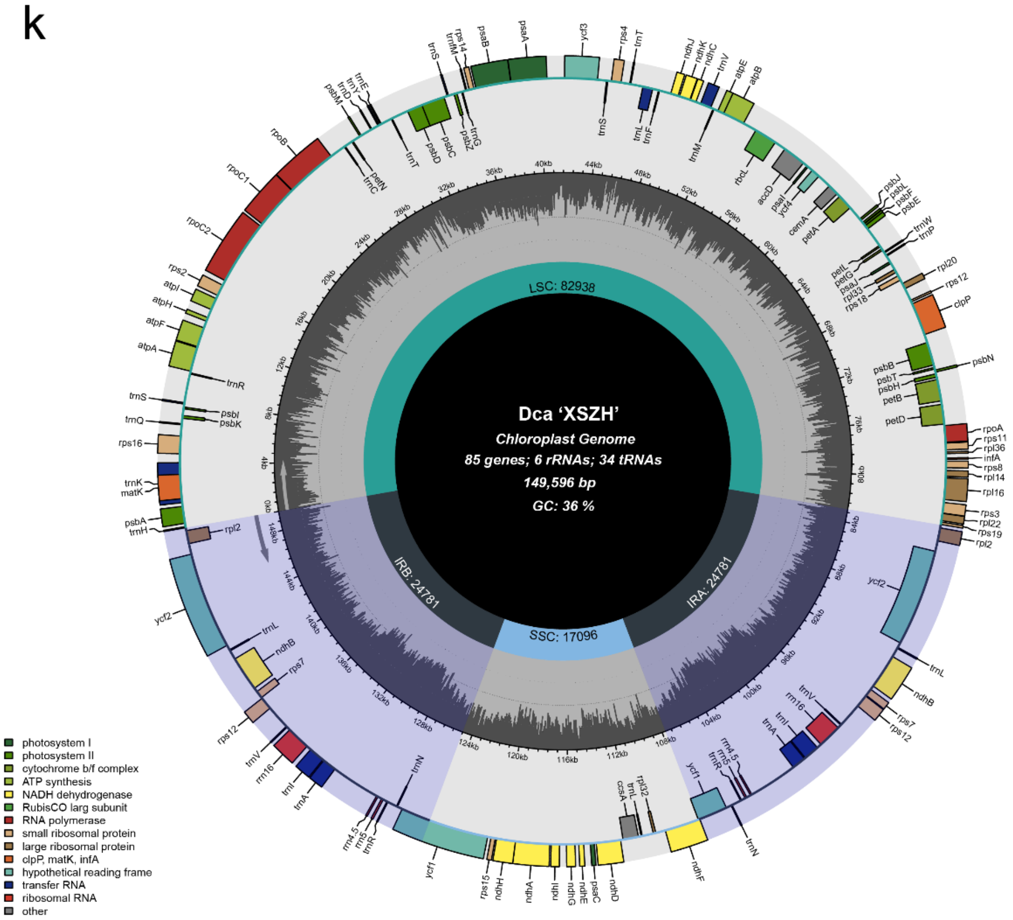
Dca 'XSZH'
Chloroplast Genome
85 genes; 6 rRNAs; 34 tRNAs
149,596 bp
GC: 36 %
LSC: 82938
IRA: 24781
SSC: 17096
IRB: 24781
photosystem I
photosystem II
cytochrome b/f complex
ATP synthesis
NADH dehydrogenase
RubisCO larg subunit
RNA polymerase
small ribosomal protein
large ribosomal protein
clpP, matK, infA
hypothetical reading frame
transfer RNA
ribosomal RNA
other

l

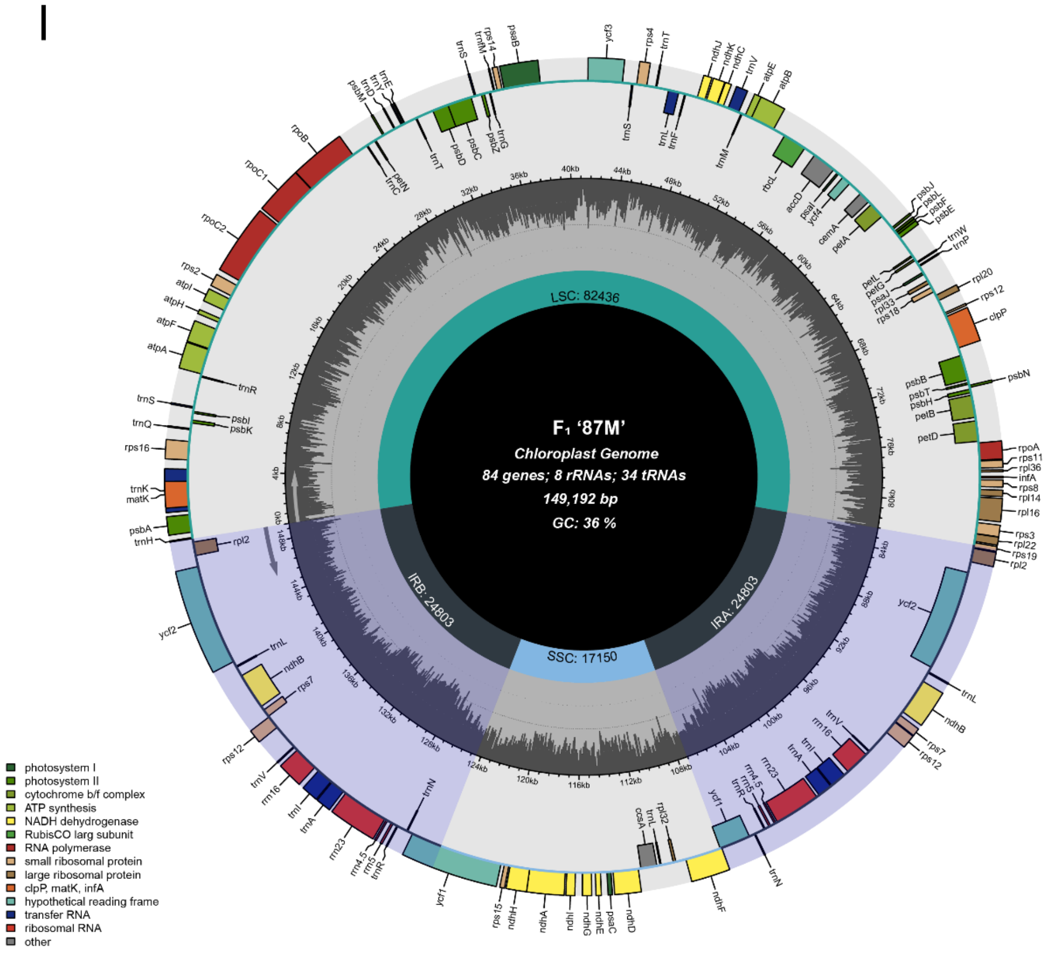

Figure S2. Gene maps of the 12 assembled *Dianthus* chloroplast genomes in this study. Genes shown inside the circle are transcribed clockwise, and those outside are transcribed counterclockwise. Different genes are color coded. The inner circle also indicates that the chloroplast genome contains a large single copy region (LSC), a small single copy region (SSC), and two copies of the inverted repeat (IRA and IRB). (a) The chloroplast genome of Dch'MH'. (b) The chloroplast genome of Dch 'dhs'. (c) The chloroplast genome of Dch 'cf'. (d) The chloroplast genome of Dch 'DPD'. (e) The chloroplast genome of Dch 'L'. (f) The chloroplast genome of Dch 'X'. (g) The chloroplast genome of 'HY'. (h) The chloroplast genome of Dsu 'QM'. (i) The chloroplast genome of 'WC'. (j) The chloroplast genome of Dbr 'XB'. (k) The chloroplast genome of Dca 'XSZH'. (l) The chloroplast genome of F_1 '87M'.

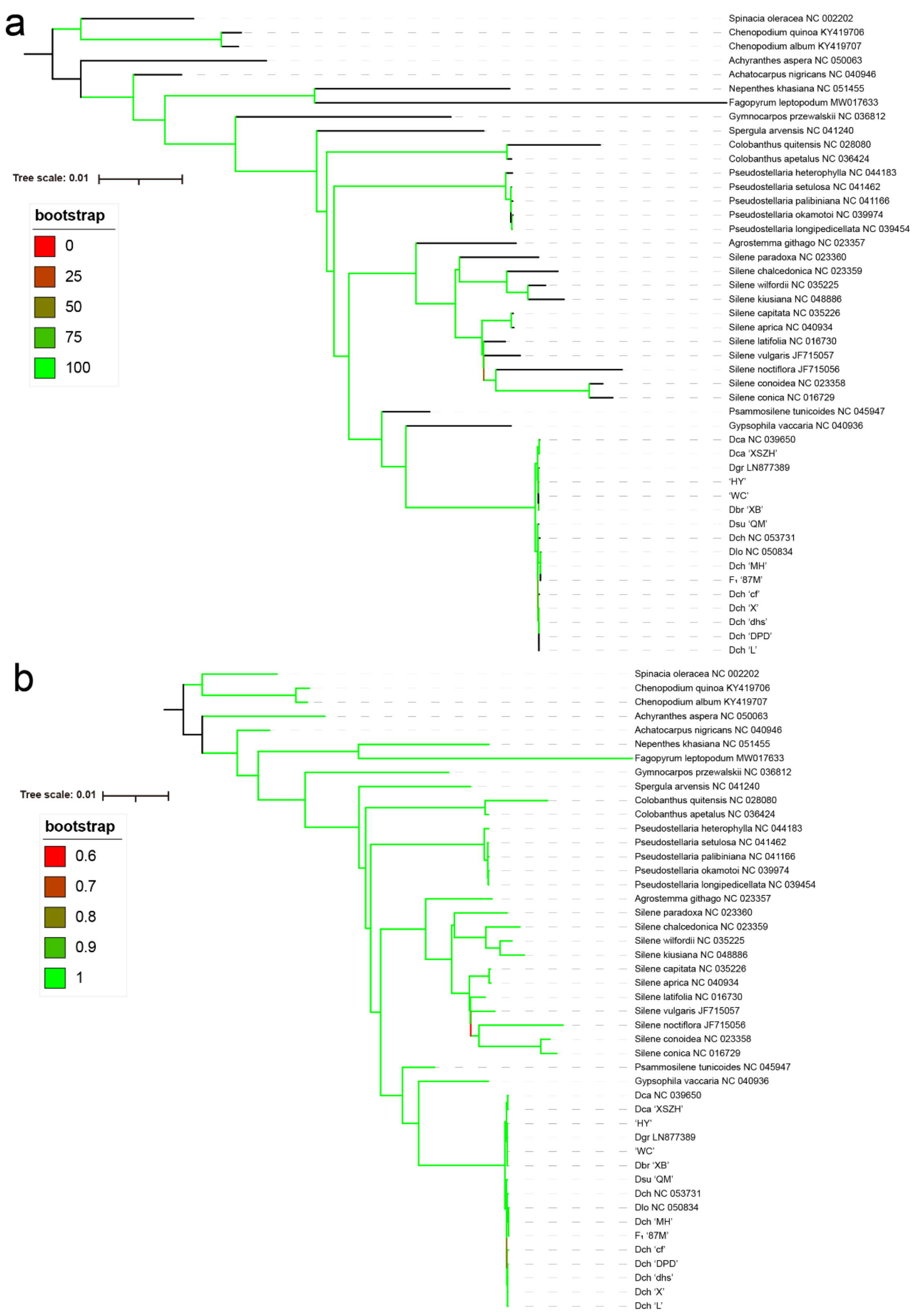
a
Tree scale: 0.01
bootstrap
0
25
50
75
100
Spinacia oleracea NC 002202
Chenopodium quinoa KY419706
Chenopodium album KY419707
Achyranthes aspera NC 050063
Achatocarpus nigricans NC 040946
Nepenthes khasiana NC 051455
Fagopyrum leptopodum MW017633
Gymnocarpos przewalskii NC 036812
Spergula arvensis NC 041240
Colobanthus quitensis NC 028080
Colobanthus apetalus NC 036424
Pseudostellaria heterophylla NC 044183
Pseudostellaria setulosa NC 041462
Pseudostellaria palibiniana NC 041166
Pseudostellaria okamotoi NC 039974
Pseudostellaria longipedicellata NC 039454
Agrostemma githago NC 023357
Silene paradoxa NC 023360
Silene chalcedonica NC 023359
Silene wilfordii NC 035225
Silene kiusiana NC 048886
Silene capitata NC 035226
Silene aprica NC 040934
Silene latifolia NC 016730
Silene vulgaris JF715057
Silene noctiflora JF715056
Silene conoidea NC 023358
Silene conica NC 016729
Psammosilene tunicoides NC 045947
Gypsophila vaccaria NC 040936
Dca NC 039650
Dca 'XSZH'
Dgr LN877389
'HY'
'WC'
Dbr 'XB'
Dsu 'QM'
Dch NC 053731
Dlo NC 050834
Dch 'MH'
F_1 '87M'
Dch 'cf'
Dch 'X'
Dch 'dhs'
Dch 'DPD'
Dch 'L'
b
Tree scale: 0.01
bootstrap
0.6
0.7
0.8
0.9
1
Spinacia oleracea NC 002202
Chenopodium quinoa KY419706
Chenopodium album KY419707
Achyranthes aspera NC 050063
Achatocarpus nigricans NC 040946
Nepenthes khasiana NC 051455
Fagopyrum leptopodum MW017633
Gymnocarpos przewalskii NC 036812
Spergula arvensis NC 041240
Colobanthus quitensis NC 028080
Colobanthus apetalus NC 036424
Pseudostellaria heterophylla NC 044183
Pseudostellaria setulosa NC 041462
Pseudostellaria palibiniana NC 041166
Pseudostellaria okamotoi NC 039974
Pseudostellaria longipedicellata NC 039454
Agrostemma githago NC 023357
Silene paradoxa NC 023360
Silene chalcedonica NC 023359
Silene wilfordii NC 035225
Silene kiusiana NC 048886
Silene capitata NC 035226
Silene aprica NC 040934
Silene latifolia NC 016730
Silene vulgaris JF715057
Silene noctiflora JF715056
Silene conoidea NC 023358
Silene conica NC 016729
Psammosilene tunicoides NC 045947
Gypsophila vaccaria NC 040936
Dca NC 039650
Dca 'XSZH'
'HY'
Dgr LN877389
'WC'
Dbr 'XB'
Dsu 'QM'
Dch NC 053731
Dlo NC 050834
Dch 'MH'
F_1 '87M'
Dch 'cf'
Dch 'DPD'
Dch 'dhs'
Dch 'X'
Dch 'L'

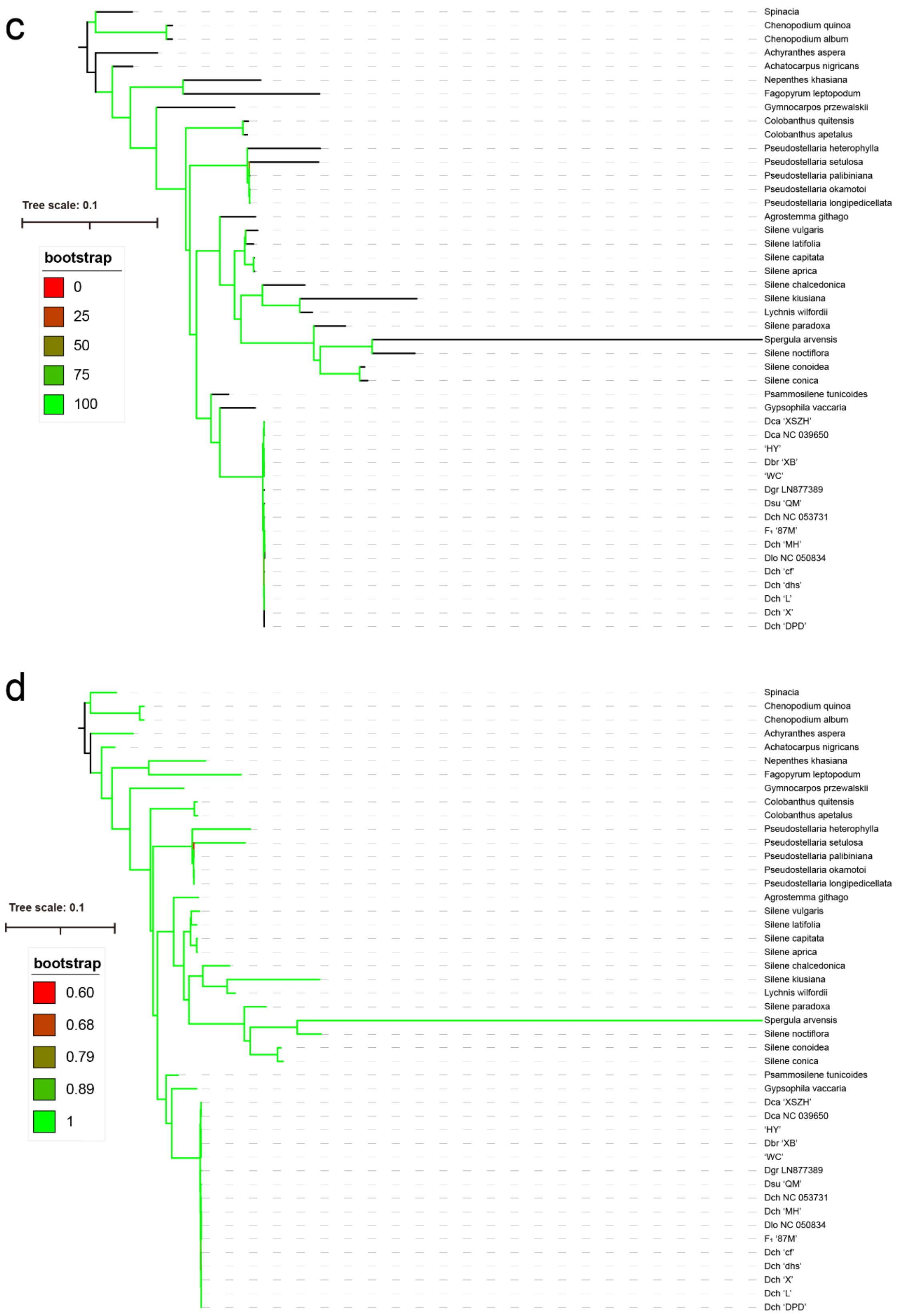
c
Tree scale: 0.1
bootstrap
0
25
50
75
100
Spinacia
Chenopodium quinoa
Chenopodium album
Achyranthes aspera
Achatocarpus nigricans
Nepenthes khasiana
Fagopyrum leptopodum
Gymnocarpos przewalskii
Colobanthus quitensis
Colobanthus apetalus
Pseudostellaria heterophylla
Pseudostellaria setulosa
Pseudostellaria palibiniana
Pseudostellaria okamotoi
Pseudostellaria longipedicellata
Agrostemma githago
Silene vulgaris
Silene latifolia
Silene capitata
Silene aprica
Silene chalcedonica
Silene kiusiana
Lychnis wilfordii
Silene paradoxa
Spergula arvensis
Silene noctiflora
Silene conoidea
Silene conica
Psammosilene tunicoides
Gypsophila vaccaria
Dca 'XSZH'
Dca NC 039650
'HY'
Dbr 'XB'
'WC'
Dgr LN877389
Dsu 'QM'
Dch NC 053731
F_1 '87M'
Dch 'MH'
Dlo NC 050834
Dch 'cf'
Dch 'dhs'
Dch 'L'
Dch 'X'
Dch 'DPD'
d
Tree scale: 0.1
bootstrap
0.60
0.68
0.79
0.89
1
Spinacia
Chenopodium quinoa
Chenopodium album
Achyranthes aspera
Achatocarpus nigricans
Nepenthes khasiana
Fagopyrum leptopodum
Gymnocarpos przewalskii
Colobanthus quitensis
Colobanthus apetalus
Pseudostellaria heterophylla
Pseudostellaria setulosa
Pseudostellaria palibiniana
Pseudostellaria okamotoi
Pseudostellaria longipedicellata
Agrostemma githago
Silene vulgaris
Silene latifolia
Silene capitata
Silene aprica
Silene chalcedonica
Silene kiusiana
Lychnis wilfordii
Silene paradoxa
Spergula arvensis
Silene noctiflora
Silene conoidea
Silene conica
Psammosilene tunicoides
Gypsophila vaccaria
Dca 'XSZH'
Dca NC 039650
'HY'
Dbr 'XB'
'WC'
Dgr LN877389
Dsu 'QM'
Dch NC 053731
Dch 'MH'
Dlo NC 050834
F_1 '87M'
Dch 'cf'
Dch 'dhs'
Dch 'X'
Dch 'L'
Dch 'DPD'

e

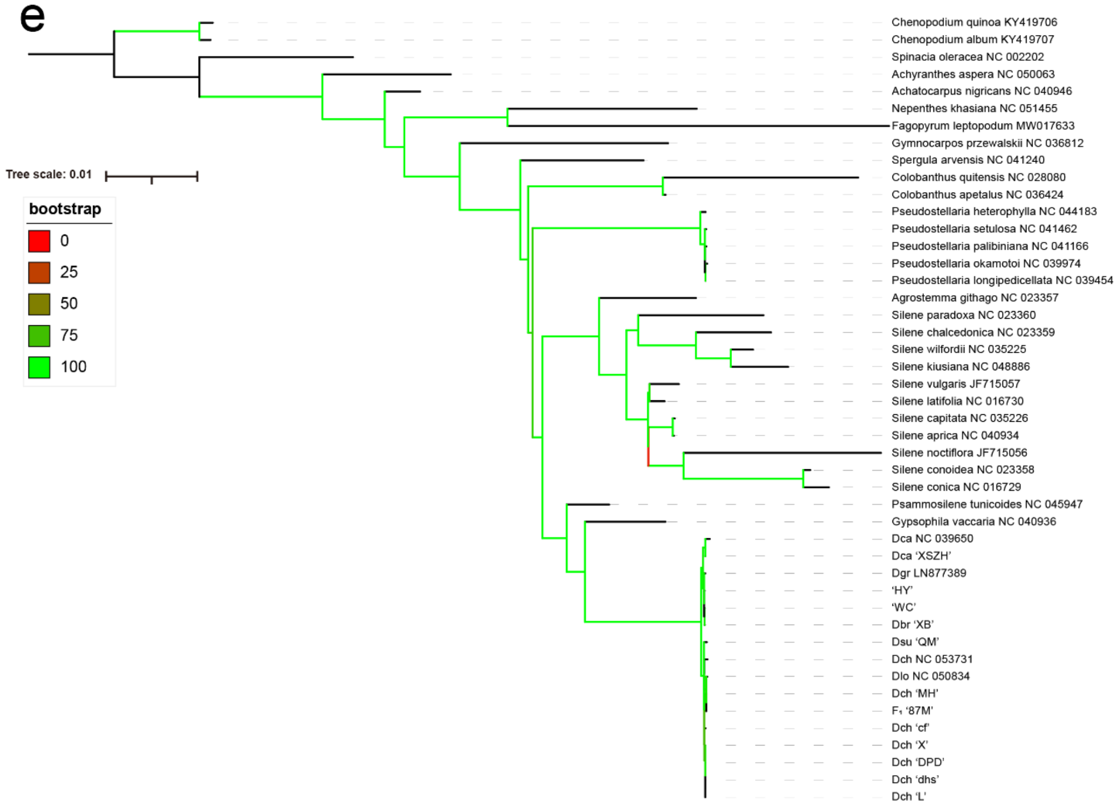

Chenopodium quinoa KY419706
Chenopodium album KY419707
Spinacia oleracea NC 002202
Achyranthes aspera NC 050063
Achatocarpus nigricans NC 040946
Nepenthes khasiana NC 051455
Fagopyrum leptopodum MW017633
Gymnocarpos przewalskii NC 036812
Spergula arvensis NC 041240
Colobanthus quitensis NC 028080
Colobanthus apetalus NC 036424
Pseudostellaria heterophylla NC 044183
Pseudostellaria setulosa NC 041462
Pseudostellaria palibiniana NC 041166
Pseudostellaria okamotoi NC 039974
Pseudostellaria longipedicellata NC 039454
Agrostemma githago NC 023357
Silene paradoxa NC 023360
Silene chalcedonica NC 023359
Silene wilfordii NC 035225
Silene kiusiana NC 048886
Silene vulgaris JF715057
Silene latifolia NC 016730
Silene capitata NC 035226
Silene aprica NC 040934
Silene noctiflora JF715056
Silene conoidea NC 023358
Silene conica NC 016729
Psammosilene tunicoides NC 045947
Gypsophila vaccaria NC 040936
Dca NC 039650
Dca 'XSZH'
Dgr LN877389
'HY'
'WC'
Dbr 'XB'
Dsu 'QM'
Dch NC 053731
Dlo NC 050834
Dch 'MH'
F1 '87M'
Dch 'cf'
Dch 'X'
Dch 'DPD'
Dch 'dhs'
Dch 'L'
Tree scale: 0.01
bootstrap
0
25
50
75
100

f

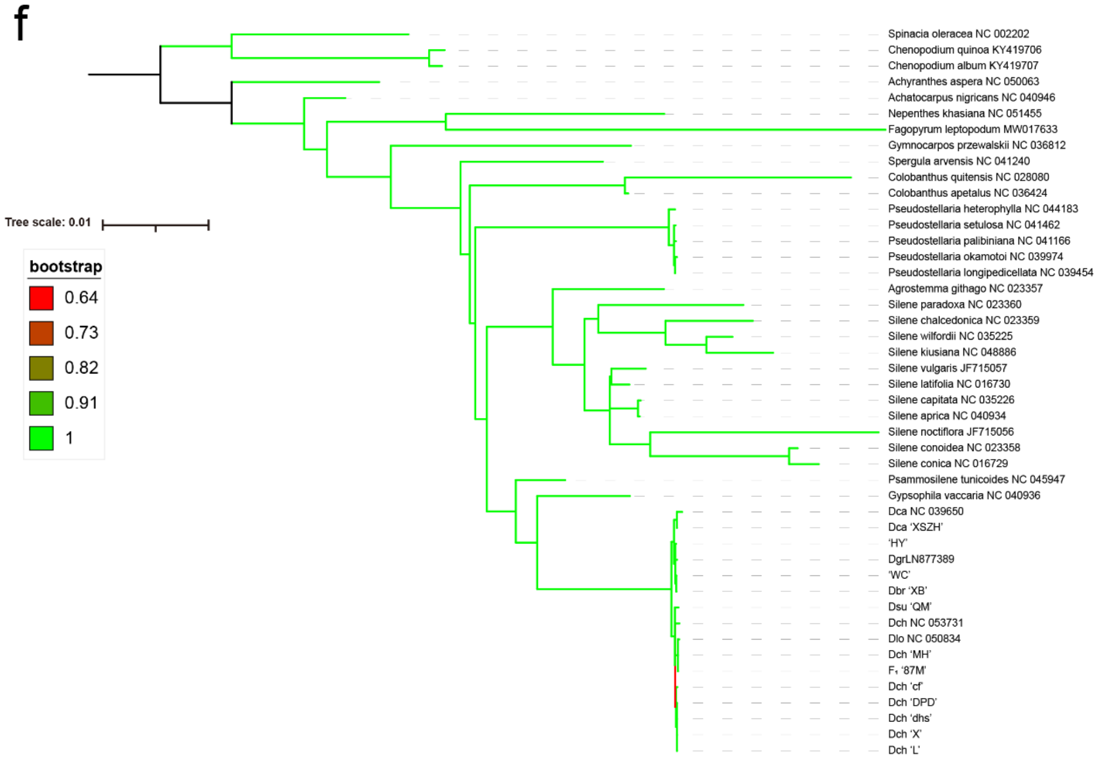

Spinacia oleracea NC 002202
Chenopodium quinoa KY419706
Chenopodium album KY419707
Achyranthes aspera NC 050063
Achatocarpus nigricans NC 040946
Nepenthes khasiana NC 051455
Fagopyrum leptopodum MW017633
Gymnocarpos przewalskii NC 036812
Spergula arvensis NC 041240
Colobanthus quitensis NC 028080
Colobanthus apetalus NC 036424
Pseudostellaria heterophylla NC 044183
Pseudostellaria setulosa NC 041462
Pseudostellaria palibiniana NC 041166
Pseudostellaria okamotoi NC 039974
Pseudostellaria longipedicellata NC 039454
Agrostemma githago NC 023357
Silene paradoxa NC 023360
Silene chalcedonica NC 023359
Silene wilfordii NC 035225
Silene kiusiana NC 048886
Silene vulgaris JF715057
Silene latifolia NC 016730
Silene capitata NC 035226
Silene aprica NC 040934
Silene noctiflora JF715056
Silene conoidea NC 023358
Silene conica NC 016729
Psammosilene tunicoides NC 045947
Gypsophila vaccaria NC 040936
Dca NC 039650
Dca 'XSZH'
'HY'
DgrLN877389
'WC'
Dbr 'XB'
Dsu 'QM'
Dch NC 053731
Dlo NC 050834
Dch 'MH'
F1 '87M'
Dch 'cf'
Dch 'DPD'
Dch 'dhs'
Dch 'X'
Dch 'L'
Tree scale: 0.01
bootstrap
0.64
0.73
0.82
0.91
1

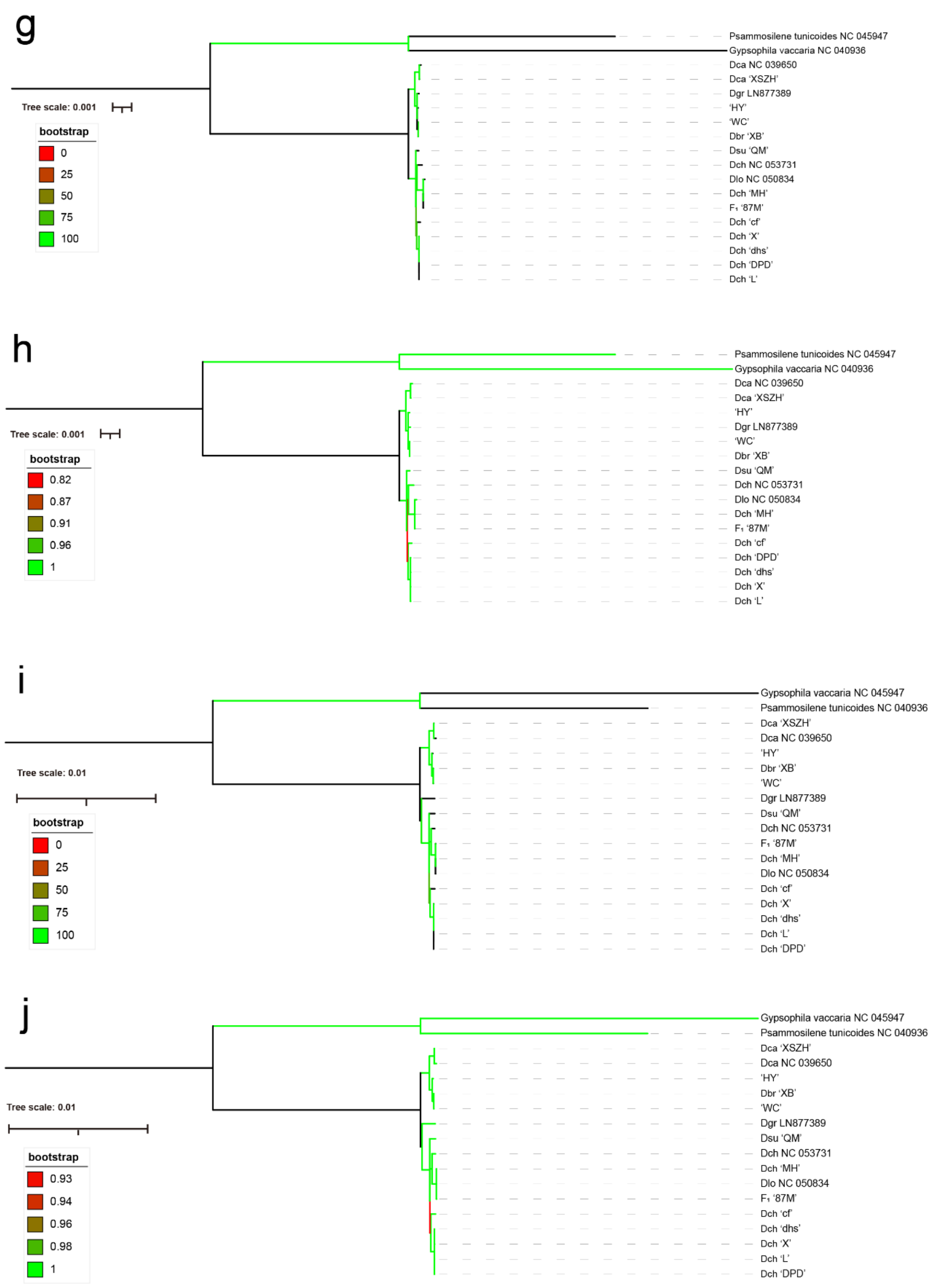
g
Tree scale: 0.001
bootstrap
0
25
50
75
100
Psammosilene tunicoides NC 045947
Gypsophila vaccaria NC 040936
Dca NC 039650
Dca 'XSZH'
Dgr LN877389
'HY'
'WC'
Dbr 'XB'
Dsu 'QM'
Dch NC 053731
Dlo NC 050834
Dch 'MH'
F_1 '87M'
Dch 'cf'
Dch 'X'
Dch 'dhs'
Dch 'DPD'
Dch 'L'
h
Tree scale: 0.001
bootstrap
0.82
0.87
0.91
0.96
1
Psammosilene tunicoides NC 045947
Gypsophila vaccaria NC 040936
Dca NC 039650
Dca 'XSZH'
'HY'
Dgr LN877389
'WC'
Dbr 'XB'
Dsu 'QM'
Dch NC 053731
Dlo NC 050834
Dch 'MH'
F_1 '87M'
Dch 'cf'
Dch 'DPD'
Dch 'dhs'
Dch 'X'
Dch 'L'
i
Tree scale: 0.01
bootstrap
0
25
50
75
100
Gypsophila vaccaria NC 045947
Psammosilene tunicoides NC 040936
Dca 'XSZH'
Dca NC 039650
'HY'
Dbr 'XB'
'WC'
Dgr LN877389
Dsu 'QM'
Dch NC 053731
F_1 '87M'
Dch 'MH'
Dlo NC 050834
Dch 'cf'
Dch 'X'
Dch 'dhs'
Dch 'L'
Dch 'DPD'
j
Tree scale: 0.01
bootstrap
0.93
0.94
0.96
0.98
1
Gypsophila vaccaria NC 045947
Psammosilene tunicoides NC 040936
Dca 'XSZH'
Dca NC 039650
'HY'
Dbr 'XB'
'WC'
Dgr LN877389
Dsu 'QM'
Dch NC 053731
Dch 'MH'
Dlo NC 050834
F_1 '87M'
Dch 'cf'
Dch 'dhs'
Dch 'X'
Dch 'L'
Dch 'DPD'

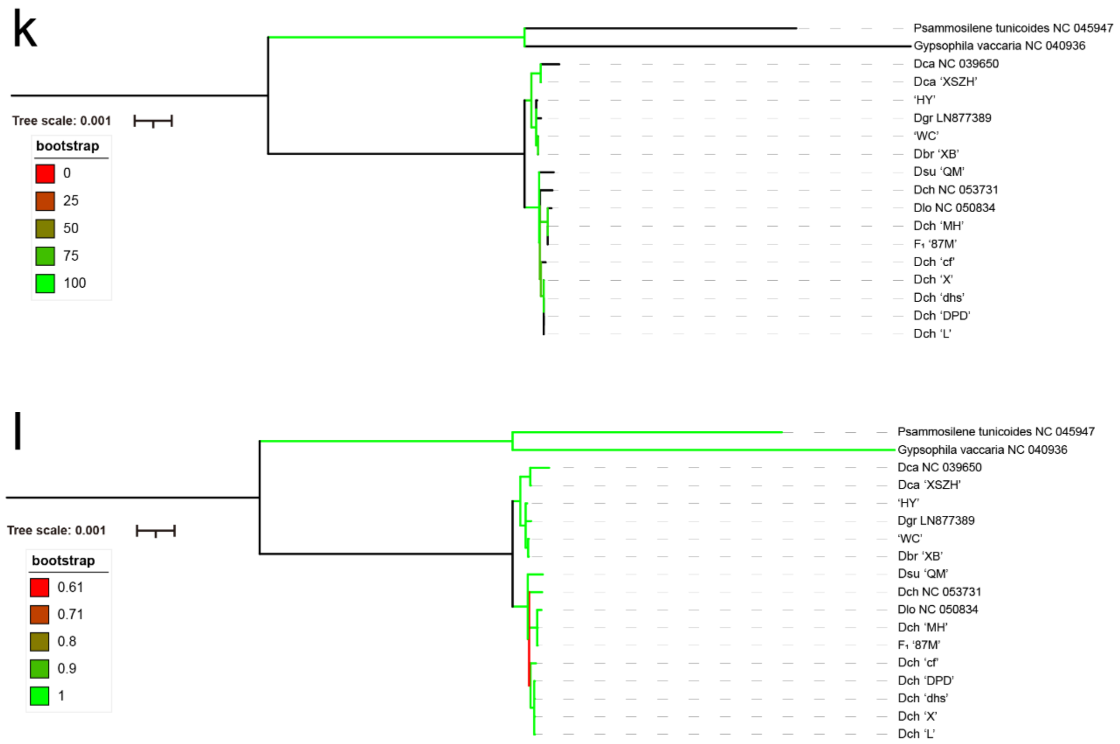

Figure S3. Phylogenetic tree based on the six datasets from Caryophyllales and *Dianthus* chloroplast genomes. (a-f) Phylogenetic tree based on the three datasets from 46 Caryophyllales chloroplast genomes. a. Phylogenetic tree based on all shared CDS from 46 Caryophyllales chloroplast genomes by maximum likelihood (ML); b. Phylogenetic tree based on all shared CDS from 46 Caryophyllales chloroplast genomes by Bayesian inference (BI); c. Phylogenetic tree based on whole chloroplast genomes from 46 Caryophyllales chloroplast genomes by maximum likelihood (ML); d. Phylogenetic tree based on whole chloroplast genomes from 46 Caryophyllales chloroplast genomes by Bayesian inference (BI); e. Phylogenetic tree based on exclude-third-codon-site matrix with deleting the terminal base of each codon from 46 Caryophyllales chloroplast genomes by maximum likelihood (ML); f. Phylogenetic tree based on exclude-third-codon-site matrix with deleting the terminal base of each codon from 46 Caryophyllales chloroplast genomes by Bayesian inference (BI). (g-l) Phylogenetic tree based on the three datasets from 16 *Dianthus* and two outgroups. g. Phylogenetic tree based on all shared CDS from 16 *Dianthus* and two outgroups by maximum likelihood (ML); h. Phylogenetic tree based on all shared CDS from 16 *Dianthus* and two outgroups by Bayesian inference (BI); i. Phylogenetic tree based on whole chloroplast genomes from 16 *Dianthus* and two outgroups by maximum likelihood (ML); j. Phylogenetic tree based on whole chloroplast genomes from 16 *Dianthus* and two outgroups by Bayesian inference (BI); k. Phylogenetic tree based on exclude-third-codon-site matrix with deleting the terminal base of each codon from 16 *Dianthus* and two outgroups by maximum likelihood (ML); l. Phylogenetic tree based on exclude-third-codon-site matrix with deleting the terminal base of each codon from 16 *Dianthus* and two outgroups by Bayesian inference (BI).

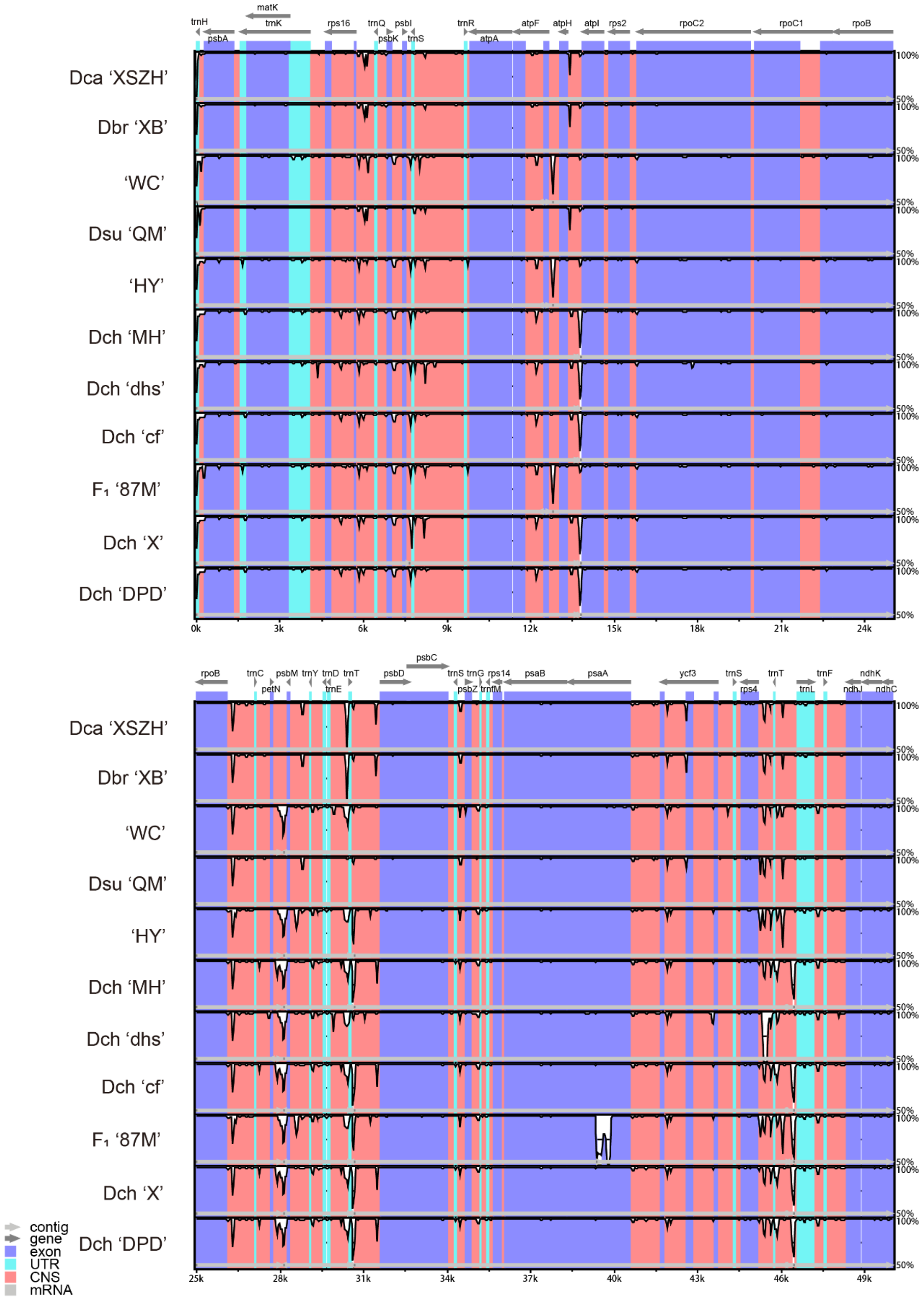
matK
trnH
trnK
rps16
trnQ
psbI
trnR
atpF
atpH
atpI
rps2
rpoC2
rpoC1
rpoB
psbA
psbK
trnS
atpA
Dca ‘XSZH’
Dbr ‘XB’
‘WC’
Dsu ‘QM’
‘HY’
Dch ‘MH’
Dch ‘dhs’
Dch ‘cf’
F₁ ‘87M’
Dch ‘X’
Dch ‘DPD’
100%
50%
0k
3k
6k
9k
12k
15k
18k
21k
24k
psbC
rpoB
trnC
psbM
trnY
trnD
trnT
psbD
trnS
trnG
rps14
psaB
psaA
ycf3
trnS
trnT
trnF
ndhK
petN
trnE
psbZ
trnfM
rps4
trnL
ndhJ
ndhC
25k
28k
31k
34k
37k
40k
43k
46k
49k
contig
gene
exon
UTR
CNS
mRNA

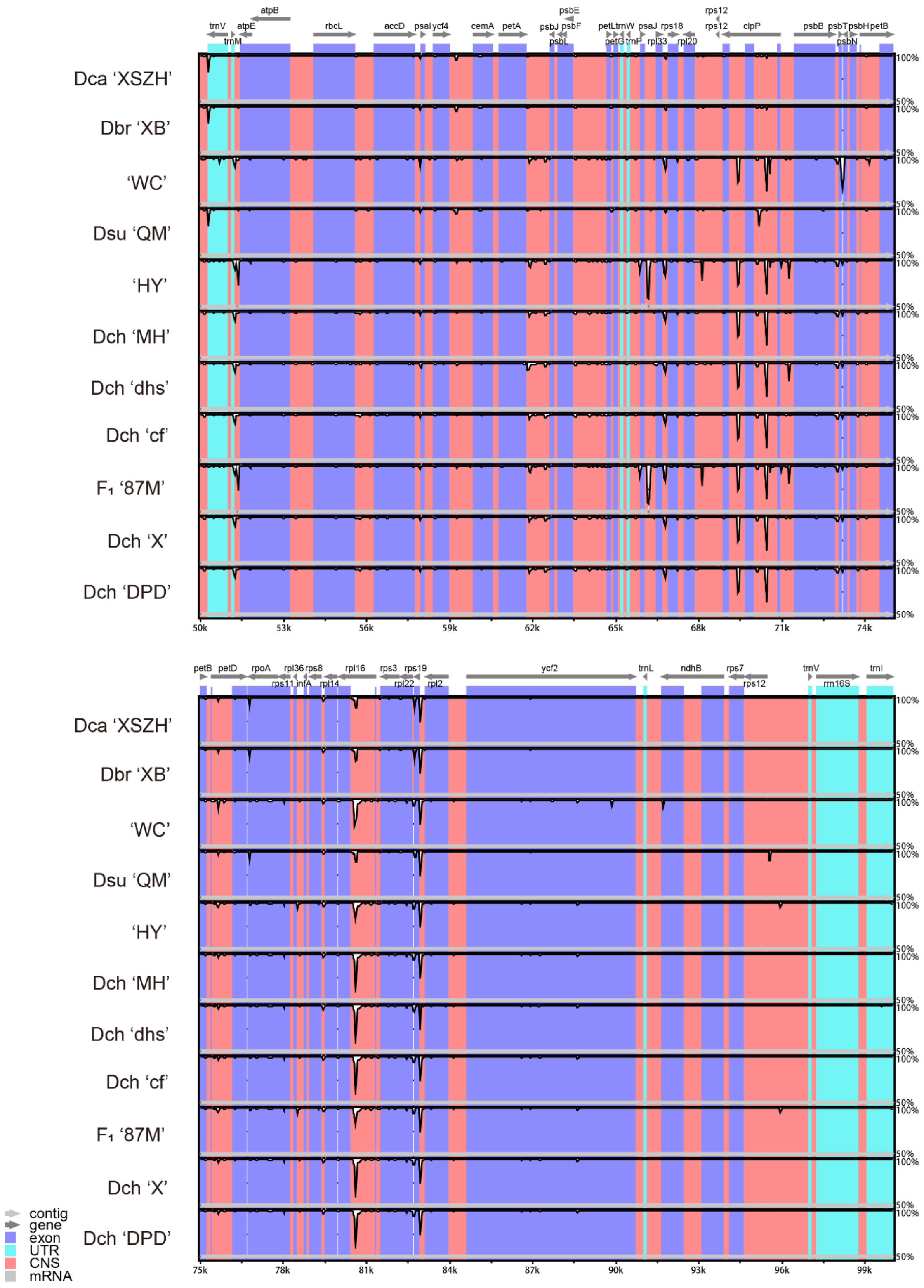
atpB
trnV atpE
trnM
rbcL
accD
psaI ycf4
cemA petA
psbE
psbJ psbF
psbL
petL trnW
petG trnP
psaJ rps18
rpl33 rpl20
rps12
rps12 clpP
psbB psbT psbH petB
psbN
Dca ‘XSZH’
Dbr ‘XB’
‘WC’
Dsu ‘QM’
‘HY’
Dch ‘MH’
Dch ‘dhs’
Dch ‘cf’
F1 ‘87M’
Dch ‘X’
Dch ‘DPD’
100%
50%
50k
53k
56k
59k
62k
65k
68k
71k
74k
petB petD rpoA rpl36 rps8 rpl16 rps3 rps19 ycf2 trnL ndhB rps7 trnV trnI
rps11 infA rpl14 rpl22 rpl2 rps12 rrn16S
75k
78k
81k
84k
87k
90k
93k
96k
99k
contig
gene
exon
UTR
CNS
mRNA

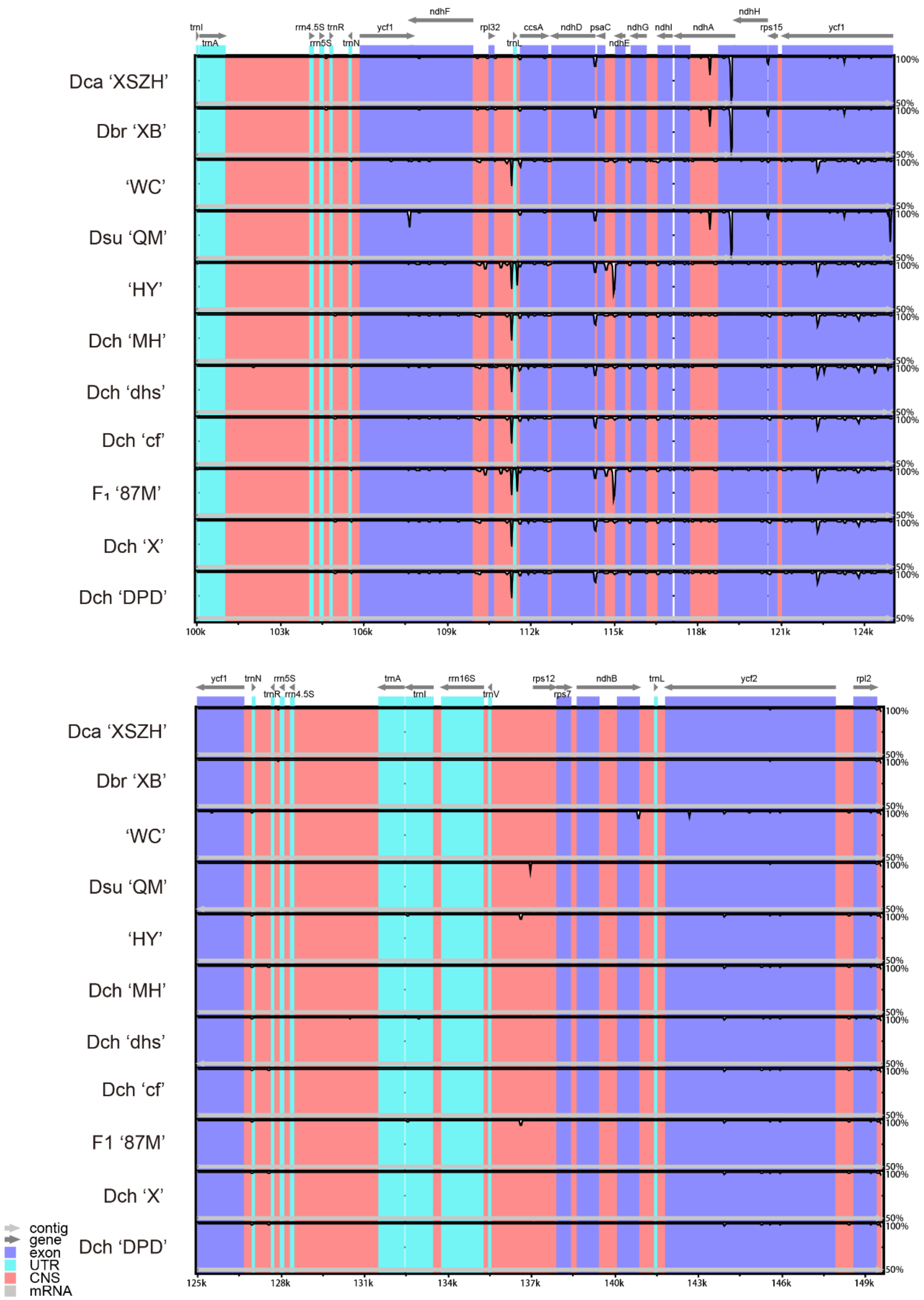

Figure S4. Sequence identity plot of the 12 *Dianthus* chloroplast genomes with 'XSZH' as a reference by mVISTA. The species names are shown to the left. The grey arrows above the alignment indicate the transcription direction of genes. In the alignment box, the blue color box indicates protein-coding, the pink color box shows the conserved noncoding sequence, and the light green box indicates tRNAs and rRNAs. The x-axis represents the positions in the chloroplast genomes, and the Y-scale represents the percent identity ranging from 50-100%.
